# MD dating: molecular decay (MD) in pinewood as a dating method

**DOI:** 10.1038/s41598-020-68194-w

**Published:** 2020-07-09

**Authors:** J. Tintner, B. Spangl, M. Grabner, S. Helama, M. Timonen, A. J. Kirchhefer, F. Reinig, D. Nievergelt, M. Krąpiec, E. Smidt

**Affiliations:** 1 Institute of Physics and Materials Science, University of Natural Resources and Life Sciences, Peter Jordan Straße 82, 1190 Vienna, Austria; 2 Institute of Statistics, University of Natural Resources and Life Sciences, Peter Jordan Straße 82, 1190 Vienna, Austria; 3 Institute of Wood Technology and Renewable Materials, University of Natural Resources and Life Sciences, Konrad Lorenz Straße 24, 3430 Tulln, Austria; 4 LUKE Natural Resources Institute Finland, Ounasjoentie 6, 96200 Rovaniemi, Finland; 5 Dendroøkologen A. J. Kirchhefer, Skogåsvegen 6, 9011 Tromsø, Norway; 6 Swiss Federal Research Institute WSL, Zürcherstrasse 111, 8903 Birmensdorf, Switzerland; 7 Faculty of Geology, Geophysics and Environmental Protection, AGH University of Science and Technology, Al. Mickiewicza 30, 30-059 Kraków, Poland

**Keywords:** Biochemistry, Statistics

## Abstract

Dating of wood is a major task in historical research, archaeology and paleoclimatology. Currently, the most important dating techniques are dendrochronology and radiocarbon dating. Our approach is based on molecular decay over time under specific preservation conditions. In the models presented here, construction wood, cold soft waterlogged wood and wood from living trees are combined. Under these conditions, molecular decay as a usable clock for dating purposes takes place with comparable speed. Preservation conditions apart from those presented here are not covered by the model and cannot currently be dated with this method. For example, samples preserved in a clay matrix seem not to fit into the model. Other restrictions are discussed in the paper. One model presented covers 7,500 years with a root mean square error (RMSE) of 682 years for a single measurement. Another model reduced to the time period of the last 800 years results in a RMSE of 92 years. As multiple measurements can be performed on a single object, the total error for the whole object will be even lower.

## Introduction

Dating of wood is important for the investigation of building histories, archaeological work, palaeoscience, and other geochronometric research. Currently, dendrochronology is the most common method used for the dating of Scots pine wood^1–7^. Radiocarbon dating is the second important dating method and is used where dendrochronology might fail for whatever reason (e.g. mainly because of a lack of long-term master reference chronologies). Higher costs and several systematic tasks must be seen as drawbacks of radiocarbon dating^8–10^. A complementary (and cheap) alternative dating approach is therefore of high interest.

Molecular decay of wood is a well-studied process. It depends on storage conditions and microbial access^11^. Fourier Transform Infrared (FTIR) spectroscopy is a well-established technique to describe and determine aging processes of organic materials^12–16^. The usefulness of molecular decay for dating purposes has already been proven in a previous work^17^. The same approach was used for this present work, namely, FTIR spectroscopy was applied and random forest models were applied for statistical evaluation. Random forest models can be seen as black box models^18^. Such an approach is useful in case of overlapping bands and complex spectral patterns. Especially the complex molecular changes during aging processes cannot easily be described in detail. Second derivatives can help to find the existence and location of overlapped and hidden peaks^19^.

Scots pine is among the most widely distributed coniferous species in the Northern Hemisphere reaching its western limits at the Iberian Peninsula and its eastern limits at the Sea of Okhotsk^20^. The postglacial reforestation history of Scots pine is quite complex. During the last glacial maximum pine species (*Pinus sylvestris*, *P. cembra*, *P. mugo*) were reduced to small patches in southern Europe, as well as in Central Europe and the Carpathian region. Other refuges were in Central Asia, like the Low Irtysh area^21^. Between 16,000 and 12,000 BP Scots pine recaptured huge areas of Southern Europe, reaching France and the Western Alps. Around 11,000 BP the north-eastern parts of continental Europe were colonized, and between 10,000 and 8,000 BP the entire Fennoscandian Peninsula was reached^22–25^. Central and Eastern Siberian regions were most likely recolonized from the region around the southern Ural^26^.

The use of Scots pine and therefore its historical and current relevance is documented by the huge number of buildings constructed more or less out of pine wood^6,27–29^. The production of tar out of birch and pine has been documented since Viking times^30^. The Sami people in northern Fennoscandia also used the inner bark as a comestible good without killing the trees^31,32^. In Alpine regions conifer needles (including from Scots pine, but predominantly from spruce) were also used for animal feed or litter in stables^33^.

The objective of this present work was the creation of a dating tool for Scots pine (*Pinus sylvestris* L.) wood covering the broad range of growth from Middle Europe to Scandinavia and including probably the oldest samples available with an age of about 14,000 years in Middle Europe and 7,500 years in northern Scandinavia. We hypothesize to find a useful model based on the molecular decay measured by means of infrared spectroscopy and evaluated by means of random forests.

## Results

### MD-model with all samples

A prediction model based on several sample sets of Scots pine wood has been built-up. Infrared spectra were measured and modelled with the dendrochronological reference. The maximal set contained 2,242 measurements on 232 wooden pieces. Sample sets and methods are given in the method section and supplementary material, respectively.

**Figure 1:**
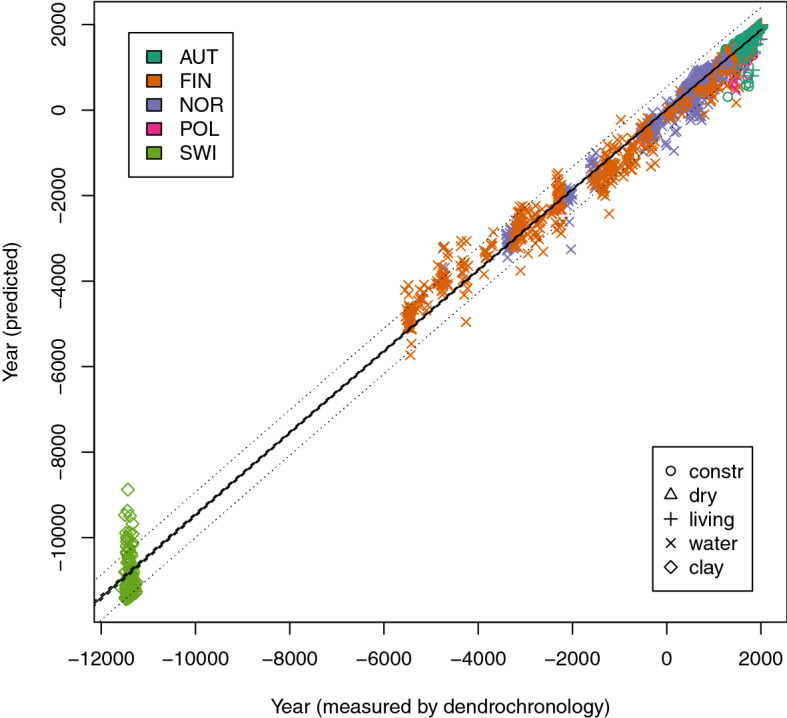
Plot of dendrochronological age and estimation results of the Random forest model based on infrared spectra. Black line indicates perfect fit, very narrow 95% confidence bands are given in dashed lines (de facto not distinguishable from perfect fit line), 95% forecast bands in dotted lines (broader intervals); different countries are indicated by different colors, different symbols indicate different preservation conditions, n = 2,242.

The molecular decay (MD)-model containing all samples revealed a similar effect as observed with the spruce model with samples from the salt environment^17^. Data are predicted with a systematic bias (Fig. 1). Waterlogged samples with an age of more than 3,000 BC are predicted above the line of perfect fit and thereby underestimated agewise. Samples around the BC/AD boundary are overestimated. This bending can be considered by a calibration step. However, as the Swiss samples from clay preservation conditions do not fit into this bending behavior they cannot be treated in a common model. We have to assume that clay preservation conditions lead to somehow different rates of molecular decay. Very specific sharp bands of clay minerals in the region between 3,700 and 3,500 cm^−1^ were missing in the spectra^34^. Therefore, we can be sure that no clay minerals entered pores or resin canals leading to spectral distortion. Moreover, apart from different preservation conditions the Swiss samples are also much older. Therefore, the comparison of the preservation conditions cannot be discussed conclusively. Further investigations and sample sets will have to be assessed for that purpose. For now the Swiss samples buried in clay deposits were taken out of the model and the modeling was repeated.

### MD-model without Swiss samples preserved in a clay matrix

**Figure 2:**
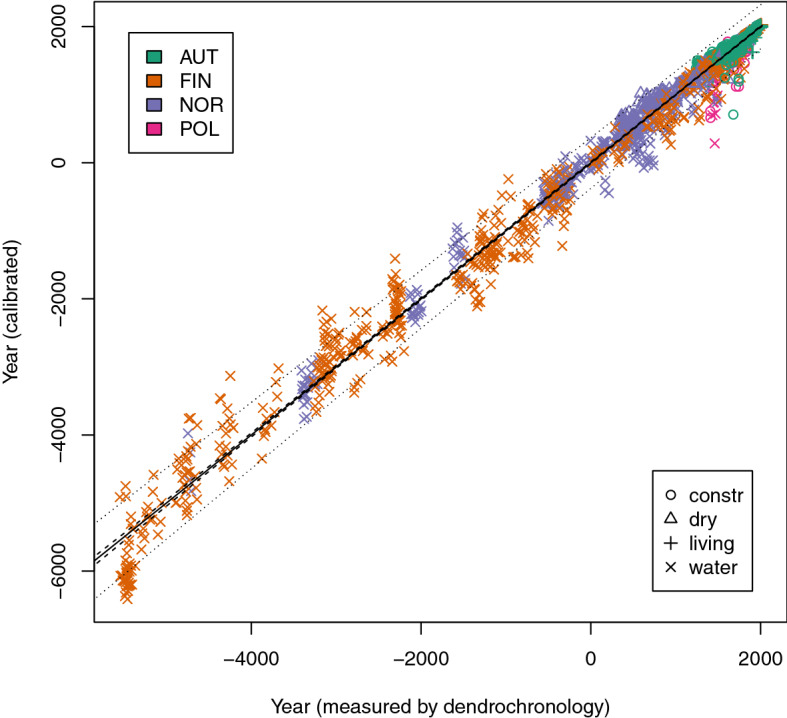
Results of the calibrated model for all samples except the Swiss samples stored in clay. Black line indicates perfect fit, very narrow 95% confidence bands are given in dashed lines, 95% forecast bands in dotted lines (broader intervals); different countries are indicated by different colors, different symbols indicate different preservation conditions, n = 2,120.

The resulting model was additionally subjected to a further calibration step according to Tintner et al.^17^. The modeling procedure used for this step is exactly the same as in the previous publication that presented models for different wood species. Furthermore, the time range is considerably extended, the spatial coverage of the samples included is far broader, and the depositional settings are more comprehensive (Fig. 2). The model quality is represented by the root mean square error and amounts to 682 years for the estimation of a single measurement. Taking into account that the model spans over 7,500 years and combines Middle European samples with samples from the Arctic zone this result can be seen as usable in many cases. Several measurements can be performed for the estimation of a certain object. An example is provided in 2.4. The results prove in particular that samples originating from Finland and Norway cannot be distinguished in terms of their prediction quality.

### MD-model with samples from AD 1,200 until the present

**Figure 3:**
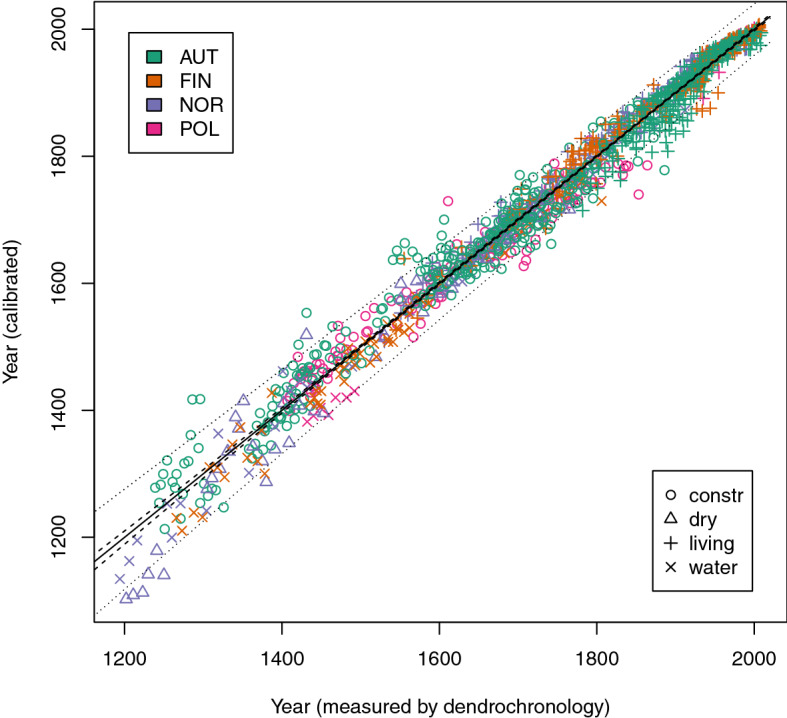
Model with samples younger than AD 1,200; Black line indicates perfect fit, very narrow 95% confidence bands are given in dashed lines, 95% forecast bands in dotted lines (broader intervals); different countries are indicated by different colors, different symbols indicate different preservation conditions, n = 1,295.

Middle European samples and samples from different preservation conditions other than waterlogged conditions are concentrated within the last 800 years. The impact of these potential influencing factors was calculated by a third model including only measurements of samples younger than AD 1,200 (Fig. 3). The model presented proves the comparability of the strongly different origins of Middle Europe and the Arctic zone. Furthermore, it proves the comparability of construction wood with cold waterlogged wood, but also with dry, cold storage in open forests. RMSE for this model amounts to 92 years.

Table 1 indicates the 30 most important wavenumbers arranged according to the four spectral regions included in the models. The wavenumbers of the spectrum reflect certain energy levels; its corresponding band height delivers information about specific molecular groups stimulated by that specific energy level. The first spectral region (2,970–2,800 cm^−1^) can be assigned to methyl and methylene groups; the second one (1,771–1,690 cm^−1^) is dominated by acetyl groups of hemicelluloses^35^. The third one (1,690–1,610 cm^−1^) can be assigned to resin acids^36^. The impact on this spectral region is relatively stronger than on the first spectral region. The last one (1,271–800 cm^−1^) contains different molecular compounds. Especially this last region contains lots of overlapping molecular vibrations from various chemical signals. In comparison to the four models presented in Tintner et al.^17^ the different pine models are more comparable to the larch model (*Larix decidua*) than to the models of silver fir, spruce or oak. This result can be expected based on the wood chemistry of the species. Interestingly, the relevance of the resin band region decreases from the shortest time span (800 years) to the longest (approx. 13,500 years). The result could be interpreted as meaning that molecular changes and probably also the gradual disappearance of resin occur predominantly in the first centuries. At least for waterlogged samples from the Arctic zone, the depletion of resin was recognized even macroscopically. The Swiss samples in particular have better resin preservation, a fact that might stress the difference in preservation conditions in a clay matrix. In any case, the prediction of age is not affected by the systematic change of a single component. As resin compounds get more and more depleted, other wood chemical compounds become more relevant for the overall prediction.

**Table 1:** Numbers of the 30 most important wavenumbers used in random forest models.

	Spectral regions (in cm^−1^)			
	2,970–2,800	1,771–1,690	1,690–1,610	1,271–800
All samples	0	12	1	17
Without clay storage	0	11	3	16
Younger than AD 1,200	0	10	4	16

### Example procedure of model usage

In order to demonstrate the model usage for the prediction of a sample, two test sets of eight randomly selected samples each were sequently excluded from the data set and the model was built up again. Based on the recorded spectral features of the test set samples, their age was predicted by the resulting model. The prediction of each measurement was corrected by the number of tree rings to the last one measured. Thereby all predictions were referenced to the last ring. Results of both test sets are combined in Table 2.

**Table 2:** Results of two test sets for validation; each line represents one sample; “observed” refers to the dendrochronological reference of the last tree ring measured by means of FTIR; “predicted mean” and “standard deviation” comprise the predictions of each measurement per sample.

Location	Preservation conditions	Number of measurements	Observed	Predicted mean	Standard deviation
AUT	Living	11	2,002	1,994	34
AUT	Constr	6	1,688	1,716	31
NOR	Water	18	− 3,210	− 1,490	632
AUT	Living	16	1,966	1,872	79
AUT	Constr	4	1,788	1,661	72
FIN	Water	11	1,560	1,470	139
FIN	Water	7	1,387	1,300	327
FIN	Water	15	1,286	1,065	266
AUT	Constr	6	1,456	1,601	158
FIN	Water	9	− 2,202	− 2,494	1,239
AUT	Living	12	2,000	2,002	31
NOR	Dry	19	1,210	1,354	162
POL	Constr	10	1,596	1,626	112
AUT	Living	19	1,982	1,935	84
AUT	Living	10	1,920	1,580	312
NOR	Living	17	1,990	1,982	50

Most of the samples are predicted well, but also some wrong predictions far apart from the reference were recorded. Future work will have to identify the reasons, why these samples do not fit properly in the model.

## Discussion

The models presented here provide a strong evidence that molecular decay can be used in a meaningful way to predict the age of Scots pine wood. They develop further what has been started by the publication of Tintner et al.^17^. In comparison to previous models, they provide several advanced insights into the applicability and usage of dating via MD. On the one hand we present a new species and in doing so we have selected a species widely found in the archaeological and historic context. Its preservation is proven to be good. Especially in the Arctic zone *Pinus sylvestris* is the only species, whose wood remains over time^37^. The impact of different climatic regions, or specific preservation conditions on the chemical decay and the resulting age determination provide a wide range of applications in different scientific fields—historical research, archaeology, and climatology. With a prediction quality of some one hundred years, MD-dating will be relevant in cases in which plenty of material is available and dendrochronology fails for any reason (for example low number of tree rings). The combination of both methods might also help to include also weak results obtained by dendrochronology into a sample set.

Results in Figs. 1 and 2 also display that within-sample variation cannot be described well by the molecular decay. Other effects randomly influence the estimated age, so vertically stacked data points can be seen in the figures. It has been proven that for young samples aging on the living tree and in wooden artefacts corresponds better (Fig. 3), but further investigations will have to separate effects of within-sample variation from aging effects driven by the preservation conditions. For practical purposes the problem is less critical: The use of MD-dating is basically based on analyzing a sample on a series of rings and takes the average as the age estimation.

Restrictions for the MD-dating tool are described in Tintner et al.^17^. Brittle parts and a half centimeter range next the outer face of the wood in construction wood cannot be used. A limited set of preservation conditions is covered by our model, namely: dry conditions of construction wood, dry conditions in open areas in a cold climate, waterlogged wood in cold soft water with a neutral pH value. Other preservation conditions have to be investigated in the future. Preservation in clay seems to affect the molecular decay changing the clock we use for our dating model. This might be explained by ion exchange effects that are in principal well known for wood^38–40^. Tintner et al.^17^ presented a different behavior of molecular decay preserved in a salty clay environment in Hallstatt, Upper Austria. We assume that corresponding effects are responsible. In order to answer that question more samples preserved in a clay matrix with ages comparable to our other samples will be necessary.

## Methods

### Material

Samples for the dating tool were taken from existing sample pools. All samples were stored in laboratories under dry conditions without any conservation agents or preservatives. Exemplary photographs are presented in Supplementary Figure S-1. Details are given in Supplementary Table S-1. The spatial distribution of samples is displayed in Supplementary Figure S-2.

#### Preservation conditions at the sampling sites

The Finnish samples comprise living tree and subfossil tree-ring materials originating from seven sites (lakes) in north-east Finnish Lapland from the counties of Utsjoki (Ailigasjärvi, Lohikoste, Vetsijärvi) and Inari (Juomusjärvi, Kompsiojärvi, Luolajärvi, Selkäjärvi). These sites were previously described in Eronen et al.^37,41^ and Helama et al.^42,43^. According to available data the pH of the lake water was near to neutral.

The Norwegian samples originated from Dividalen, an intra-alpine valley in inner Troms, northern Norway. The nearest meteorological station recorded an annual precipitation of 282 mm. Living trees represented dominant, solitary individuals growing at 370–420 m a.s.l. on the south-facing slope of Mt. Skrubben/Lulit Čavárri^44^. Dry preservation covers samples from lying trunks and coarse wood debris on dry forest ground between the valley bottom at 220 m a.s.l. and the pine tree line at 470 m a.s.l. mainly close to the creek Sleppelva^45^. Most waterlogged samples originated from subfossil logs in the lake Brennskogtjønna (312 m a.s.l., 250 m × 575 m). Three samples originate from a nearby small lake (90 m × 90 m) at 405 m a.s.l., described as Gauptjønna in Jensen et al.^46^. The pH of both lakes might be affected by calcareous bedrock.

The Swiss subfossil samples originated from the north-eastern flank of Uetliberg in Zurich, Switzerland. Covered in an up to eight-meter-deep homogenous clay package, the excavated in situ stumps have been well preserved under undisturbed, anaerobic conditions. They were covered by clay and were found at a depth of eight meters^47^. After discovery the samples were dried, leading to increased cell decomposition especially within the outer sapwood.

The Austrian construction wood originated from six different sites (castles and churches) in the two regions Waldviertel and Weinviertel of the Austrian state of Lower Austria. The recent samples originated from two different sites, one in each of these regions^48–50^.

The Polish samples originated from seven churches situated along an N-S transect through Poland from the Baltic Sea to the southern border with the Czech Republic and Austria. Another analyzed object was a wooden water pipe discovered and lifted at archaeological excavations in Pleszew (central Poland). Living trees originated from two research sites—forests Włocławek and Ojców National Park.

### Methods

#### Dendrochronological reference

All samples were collected and analyzed during previous field campaigns within the last 25 years and dated according to standard procedures^50–52^. The Finnish samples were dendrochronologically cross-dated against the existing Scots pine tree-ring chronologies from the same region^4,37,42,43,53^. The Polish samples were cross-dated against the existing Scots pine chronologies from the same region^54,55^. The Swiss samples were selected from a floating Zurich Late Glacial ring width chronology, containing more than 300 trees, which have been dated through radiocarbon measurements. The Austrian samples were cross-dated against the regional pine-chronologies (Waldviertel and Weinviertel).

#### Fourier Transform Infrared (FTIR) spectroscopy

FTIR spectra were recorded in the ATR (attenuated total reflection) mode in the mid infrared area (4,000–400 cm^−1^) with an optical crystal of a BRUKER Helios FTIR micro sampler (Tensor 27). This device allows spot measurements with a spatial resolution of 250 microm. 32 scans were collected at a spectral resolution of 4 cm^−1^. Spectra were vector normalized using the OPUS (version 7.2) software. Smoothing and second derivative spectra were obtained using The Unscrambler X 10.1 (CAMO) by applying the Savitzky–Golay algorithm^56^. Only the smoothed second derivative spectra were further processed.

#### Statistical methods

The model was established in the same way as the models presented in Tintner et al.^17^. The same band regions of the model for larch (*Larix decidua*) were used: 2,970–2,800 cm^−1^, 1,771–1,690 cm^−1^, 1,690–1,610 cm^−1^, 1,270–800 cm^−1^. Additionally the band from 880 to 865 cm^−1^ has been kept out of analyses. This band is assigned to calcite^57^ originating from remnants of chalk that was used to make tree rings more visible. All statistical analysis was done using the statistical computer software language R^58^. The R package randomForest^59^ was used to fit a random forest model to the data. Models were tenfold cross-validated.

## Supplementary information

Supplementary file1 (DOCX 5887 kb)

Supplementary file2 (CSV 41366 kb)

## Data Availability

All code and result data in this study to perform the analyses and to create the figures can be made available upon request to the corresponding author. Original data are provided as supplementary material.
