# Supplementary material for: MD dating: molecular decay (MD) in pinewood as a dating method
Source: Sci Rep. 2020 Jul 9;10:11255. doi: 10.1038/s41598-020-68194-w (PMC7347527; doi:10.1038/s41598-020-68194-w)
Supplement: Supplementary file 1 — Supplementary file1 (DOCX 5887 kb) [file 41598_2020_68194_MOESM1_ESM.docx]

**MD Dating – Molecular decay (MD) in pinewood as a dating method**

Tintner J., Spangl B., Grabner M., Helama S., Timonen M., Kirchhefer A.J., Reinig F., Nievergelt D., Krąpiec M., Smidt E.

**d**

**b**

**a**

**c**


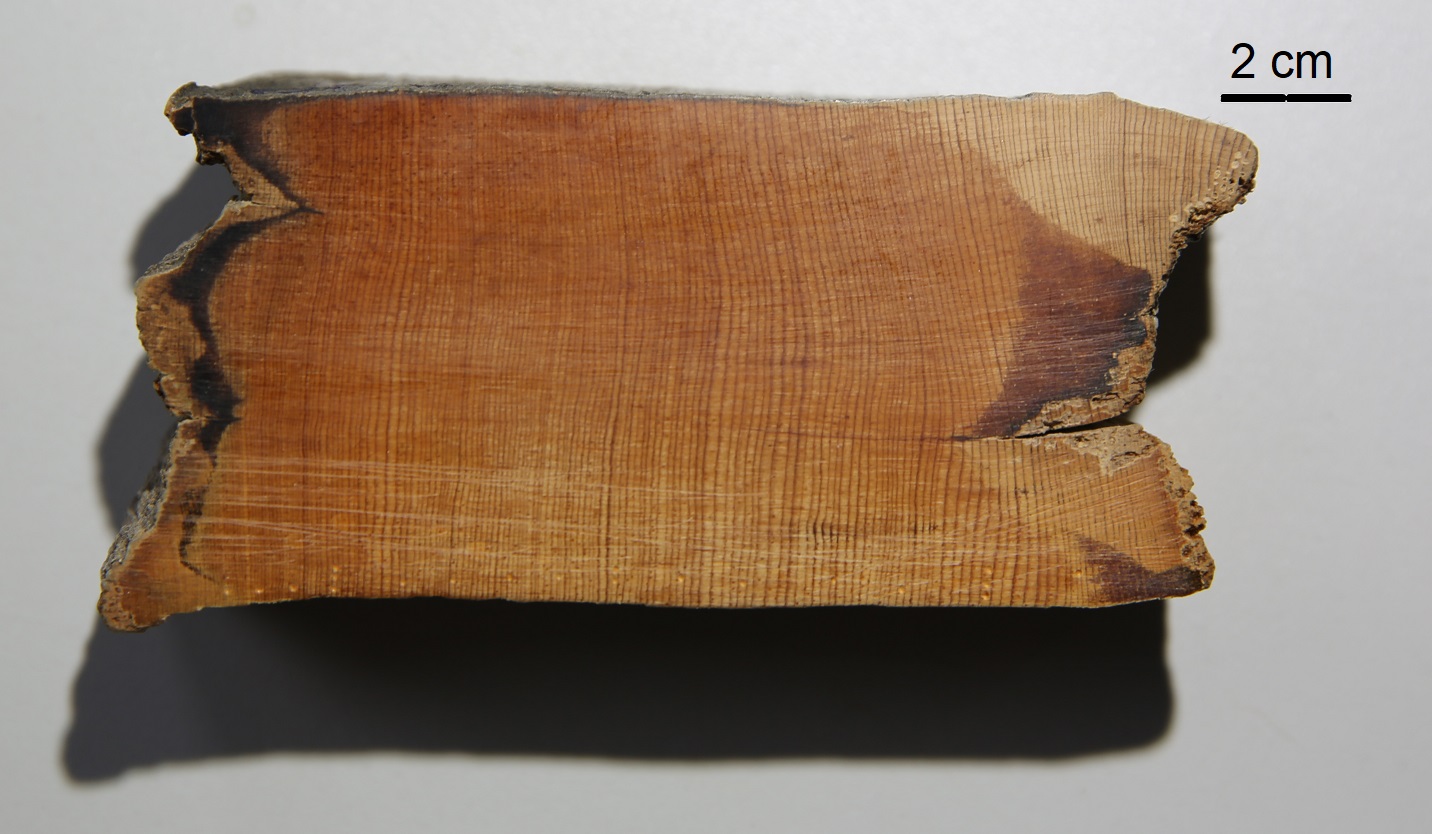

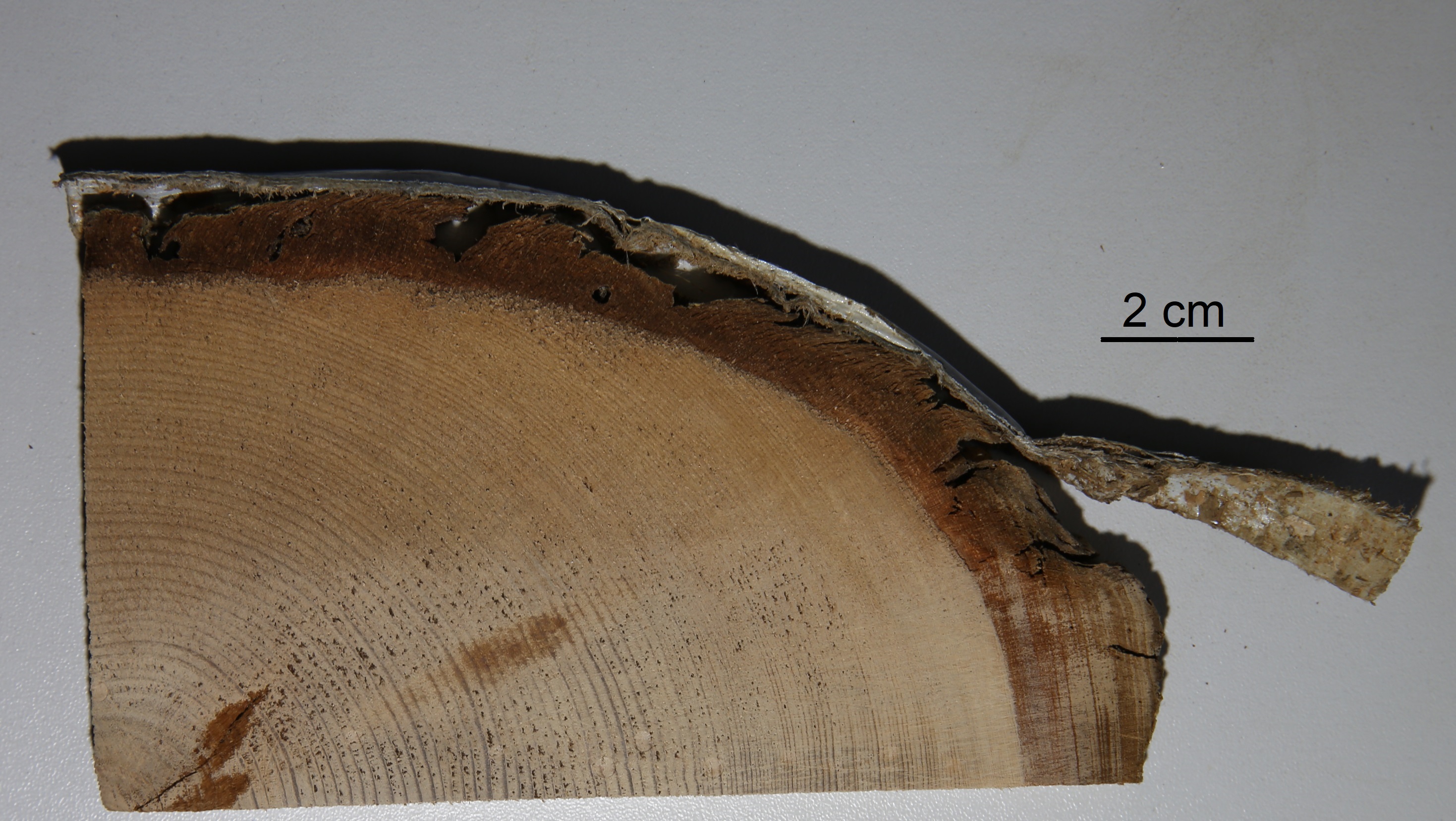

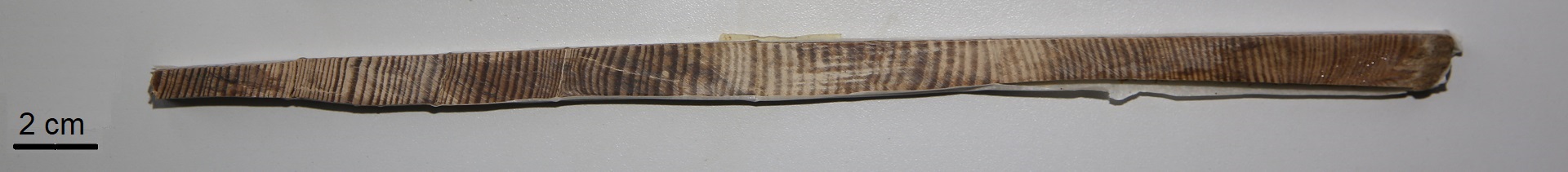

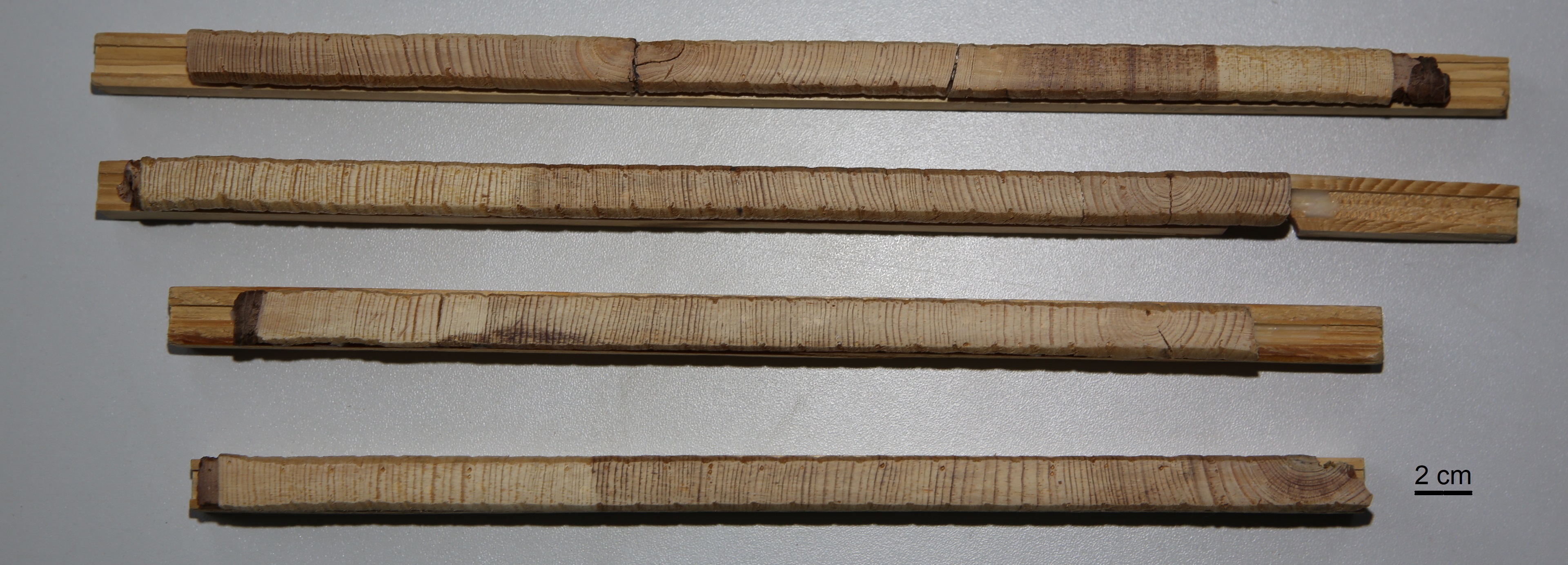


Supplementary Figure S-1: Exemplary photos of samples stored under different conditions; a: dry (Norway); b: waterlogged (Finland); c: clay (Switzerland); d: 4 cores from living trees (Norway); black bars represent 1 cm


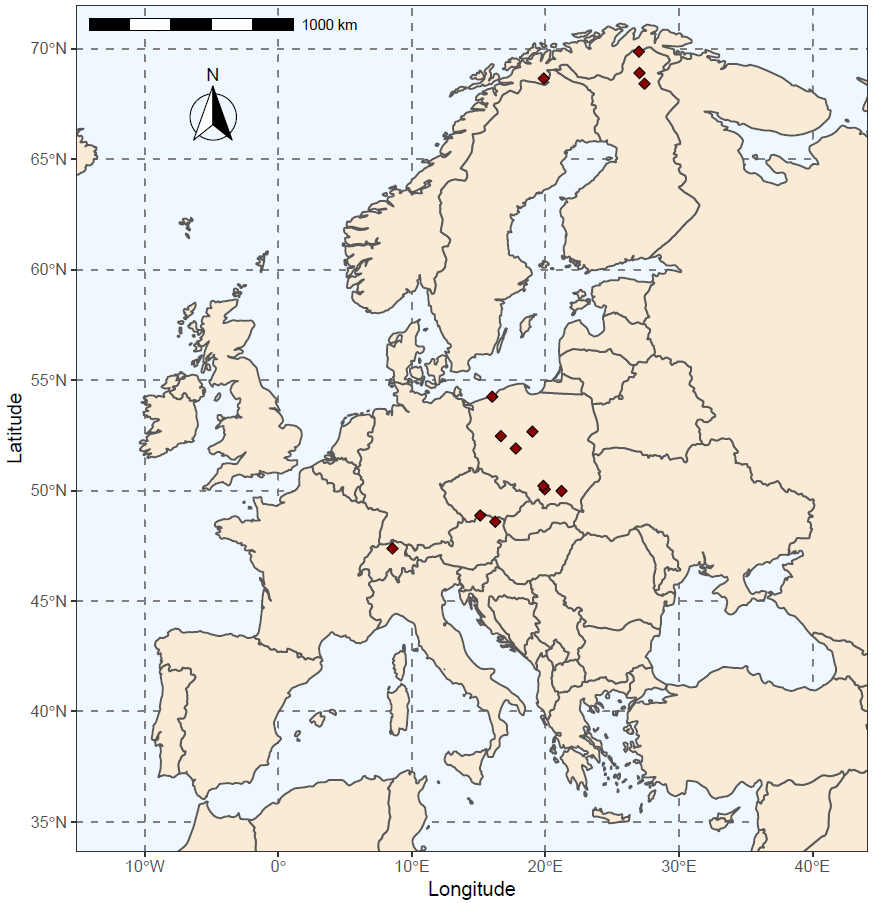


Supplementary Figure S-2: Sample distribution, sampling sites marked with red diamonds; Additionally to the software R 4.0.1 (https://www.R-project.org) the R package "rnaturalearthdata" (version 0.1.0) under license CC0 (https://creativecommons.org/publicdomain/zero/1.0/legalcode) was used providing vector map data from http://www.naturalearthdata.com

**Supplementary Table S-1:** Origin, preservation conditions and number of samples and measurements for the dating tool

| location | preservation conditions | time span  (years AD) | no. of samples | no. of measurements |
| --- | --- | --- | --- | --- |
| Finland | living | 1545 to 2008 | 11 | 143 |
|  | waterlogged | -5549 to 1806 | 53 | 515 |
| Norway | living | 1524 to 1991 | 9 | 165 |
|  | dry | 327 to 1765 | 17 | 169 |
|  | waterlogged | -4747 to 1912 | 31 | 292 |
| Switzerland | clay | -11605 to -11252 | 10 | 122 |
| Austria | living | 1782 to 2009 | 21 | 263 |
|  | construction wood | 1239 to 1936 | 62 | 368 |
| Poland | living | 1910 to 2008 | 5 | 56 |
|  | waterlogged | 1432 to 1492 | 1 | 7 |
|  | construction wood | 1407 to 1864 | 12 | 142 |
| sum |  | -11605 to 2009 | 232 | 2,242 |
